# Power-to-Noise Optimization in the Design of Neural Recording Amplifier Based on Current Scaling, Source Degeneration Resistor, and Current Reuse

**DOI:** 10.3390/bios14020111

**Published:** 2024-02-19

**Authors:** Zhen Wang, Xiao Wang, Guijun Shu, Meng Yin, Shoushuang Huang, Ming Yin

**Affiliations:** 1 State Key Laboratory of Digital Medical Engineering, School of Biomedical Engineering, Hainan University, Haikou 570100, China; zhen_wang@hainanu.edu.cn (Z.W.); wangx@hainanu.edu.cn (X.W.); shugj@hainanu.edu.cn (G.S.); myin@hainanu.edu.cn (M.Y.); shoushuanghuang@hainanu.edu.cn (S.H.); 2 Key Laboratory of Biomedical Engineering of Hainan Province, One Health Institute, Hainan University, Haikou 570100, China

**Keywords:** neural signal amplifier, current scaling, source degeneration resistors, current reuse

## Abstract

This article presents the design of a low-power, low-noise neural signal amplifier for neural recording. The structure reduces the current consumption of the amplifier through current scaling technology and lowers the input-referred noise of the amplifier by combining a source degeneration resistor and current reuse technologies. The amplifier was fabricated using a 0.18 μm CMOS MS RF G process. The results show the front-end amplifier exhibits a measured mid-band gain of 40 dB/46 dB and a bandwidth ranging from 0.54 Hz to 6.1 kHz; the amplifier’s input-referred noise was measured to be 3.1 μVrms, consuming a current of 3.8 μA at a supply voltage of 1.8 V, with a Noise Efficiency Factor (NEF) of 2.97. The single amplifier’s active silicon area is 0.082 mm^2^.

## 1. Introduction

The emerging field of Brain–Machine Interface (BMI) technology utilizes microelectrodes, microelectronics, and computational technologies and has extensive applications in neural research and neuroscience [1]. Advanced microelectromechanical systems (MEMS) technology allows for the integration of multiple neural microelectrode systems onto a single silicon chip [2], which can then be implanted into the cerebral cortex. Such systems can simultaneously capture full-spectrum neural signals from multiple neurons. The subsequent analysis of these neural signals allows for the establishment of a connection between neural responses and real bodily activities, thereby facilitating brain–machine control [3]. Consequently, neural recording amplifiers play a crucial role in the development of BMI technology and are considered an indispensable component.

The electrochemical effects at the electrode–tissue interface often lead to a DC offset of 1–2 V in differential recording electrodes [4]. Therefore, the electrodes need to be AC coupled to the amplifiers to eliminate this offset. Local Field Potentials (LFPs), which are neural signals, typically exhibit amplitudes ranging from 20 μV to 1 mV, covering a frequency range of 1 Hz to 200 Hz. In contrast, Action Potentials (APs) generally have an amplitude of around 50 μV, but they can reach as high as 5 mV in cases of abnormal multi-unit activity; these signals can have a frequency content of up to 5 kHz [5], and occasionally, even higher.

Because neural signals have a low amplitude, noise and interference can significantly affect the recorded signals. Maintaining a low input-referred noise in the amplifier is crucial for obtaining clean neural signal recordings. Technologies commonly used to reduce the input-referred noise in amplifiers include source degeneration resistors [6], current reuse [7,8], and g_m_-boost [9]. In fact, during the process of signal acquisition, the thermal and biological background noise are typically around 10 μVrms [10]. Therefore, within the amplifier’s −3 dB bandwidth, it is crucial to keep the input-referred noise of the amplifier below the background noise level.

Besides input-referred noise, implantable bioamplifiers need to operate with low power consumption to prevent thermal damage to the surrounding neural tissue. Implanted systems that dissipate more than 40 mW of power can result in a temperature increase of over 2 °C, which can lead to cell death within a few days [11]. To ensure the safety of the tissue, it is recommended to limit the power consumption per channel to the range of 25–50 µW, effectively restricting power dissipation and ensuring that the tissue heating remains below 1 °C [12]. This requirement is especially critical for multi-channel neural recording systems, where low power consumption is essential. Additionally, amplifiers should have a wide −3 dB bandwidth to capture a broader range of signals. Thus, in the design of neural signal amplifiers, achieving a balance between power consumption, noise, and −3 dB bandwidth is crucial. To compare the noise–power trade-off among amplifiers, we adopt the NEF proposed in [4], which is widely used for evaluating the noise–power trade-off in neural amplifier designs.

There have been many excellent research efforts aimed at reducing the NEF to address the trade-off between amplifier noise and power consumption [6,13,14,15,16]. However, these endeavors often face challenges in finding a trade-off among noise, power consumption, and the amplifier’s −3 dB bandwidth.

This paper presents a novel amplifier architecture that combines current scaling, a source degeneration resistor, and current reuse technologies to effectively balance the power consumption, noise, and −3 dB bandwidth of the amplifier. This design aims to ensure low noise and low power consumption while achieving a wide bandwidth range. Measurements indicate that the single-channel amplifier consumes 6.84 μW of power, has an input-referred noise of 3.1 μVrms in the 1 Hz–6.1 kHz bandwidth, a PSRR (Power Supply Rejection Ratio) and CMRR (Common Mode Rejection Ratio) at 1 kHz of 84 dB and 66 dB, respectively, and a −3 dB bandwidth ranging from 0.54 Hz to 6.1 kHz.

The organizational structure of this paper is as follows: Section 2 introduces the overall system architecture of the capacitively-coupled instrumentation amplifier (CCIA), Section 3 discusses the proposed low-power, low-noise Operational Transconductance Amplifier (OTA) structure, Section 4 presents the detailed circuit implementation, Section 5 presents the measurement results, and the conclusion is provided in Section 6.

## 2. Overall System Architecture

**Figure 1:**
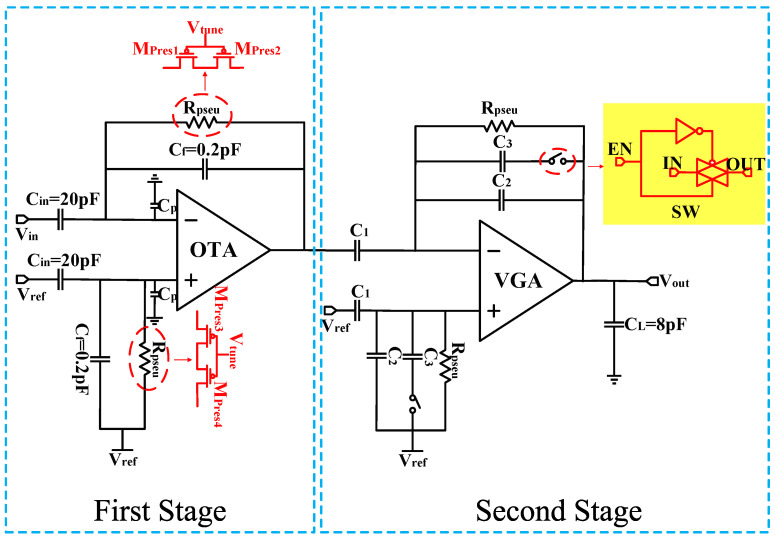
Overall schematic of the neural amplifier.

The classic approach to implementing the front end of neural recording is widely adopted in closed-loop CCIA structures [5,6,7,8,9,13,14,15,17]. The typical circuit structure is shown in Figure 1.

In this first stage of the schematic, the input signals are AC coupled through a pair of input capacitors (C_in_), and a negative feedback network formed by a feedback capacitor (C_f_) is applied around the OTA for operation. Hence, the closed-loop gain of the amplifier is defined by the ratio of C_in_/C_f_. The lower cutoff frequency (f_L_) is given by 1/(2πR_pseu_C_f_), while the higher cutoff frequency (f_H_) is given by g_m_/(2πC_L_), where g_m_ represents the transconductance of the OTA, and R_pseu_ is the pseudoresistor formed by the PMOS transistors. One advantage of this design is its ability to occupy a small area while exhibiting resistance characteristics of over 100 GΩ within a voltage difference of less than ±0.2 V [17]. Additionally, the resistance value of the pseudo-resistor can be adjusted by an external voltage V_tune_, allowing for tunable cutoff frequencies.

The calculation formula for the input-referred noise of the amplifier is as follows:

(1) $$\bar{{V^{2}}_{AMP}}={\left(\frac{C_{\mathrm{in}}+C_{f}+C_{p}}{C_{\mathrm{in}}}\right)}^{2}\bar{{V^{2}}_{OTA}}$$

where $\bar{{V^{2}}_{AMP}}$ is the input-referred noise of the amplifier, $\bar{{V^{2}}_{OTA}}$ is the input-referred noise of the OTA, C_p_ is the parasitic capacitance within the OTA.

According to Equation (1), to achieve a low-noise amplifier, it is essential to ensure that the input capacitance C_in_ >> C_f_, C_p_.

The second stage of the schematic is a Variable Gain Amplifier (VGA). The VGA is based on a CCIA topology as well, and offers two different programmable gains which are set via a programmable capacitor array. Therefore, the total gain of the amplifier can be set to ×200 and ×100.

In addition, due to the significantly lower gain of the VGA in comparison to the gain of the first-stage, the influence of the VGA on the overall amplifier’s input-referred noise is correspondingly negligible. Hence, to achieve low-noise performance, it is important to design the first-stage OTA to have low input-referred noise. Section 3 describes the low-noise low-power design technologies used in the OTA.

## 3. The Proposed Low-Power, Low-Noise OTA

### 3.1. Proposed OTA

**Figure 2:**
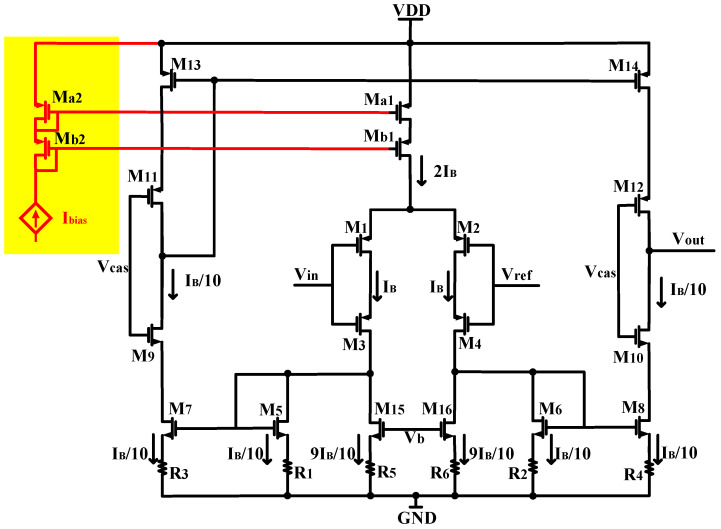
Circuit diagram of the low-power, low-noise OTA used in this design.

In the OTA depicted in Figure 2, to achieve a 1:10 current scaling and reduce circuit power consumption, we apply a bias voltage V_b_ to M_15_ and M_16_. This bias voltage sets the current flowing through M_15_ and M_16_ at 9/10 I_B_. Consequently, the current of the branch transistor M_5_–M_8_ is configured to be 1/10 I_B_. This approach enables current scaling in the circuit without requiring additional bias current consumption. The self-biased structure eliminates any additional current consumption from the individual branches that provide bias and removes the necessity for complex circuits to supply the bias voltage to the amplifier. As a result, the operating conditions of the amplifier are simplified. Furthermore, to optimize the noise of the amplifier, we employed source degeneration resistors with identical resistance values and the current mirror transistors M_5_–M_8_ are identical while the size of M_15_–M_16_ are also identical to mitigate matching errors that could occur when using source degeneration current mirrors with different sizes. The previous approaches to achieve current scaling involved utilizing source degeneration current mirrors with different sizes at the bottom [6,13] to regulate the current replication ratio of source degradation current. However, in the actual manufacturing process, variations and process errors can introduce matching errors when using different sizes of source degeneration current mirrors. This can result in inaccurate current replication ratios and increase the risks of equipment mismatch. Therefore, the use of different sizes of source degeneration current mirrors carries a higher risk of errors and can lead to increased equipment mismatch. To mitigate these risks, employing source degeneration current mirrors with the same size can help reduce matching errors and enhance the overall performance and reliability of the equipment.

To minimize the input-referred noise of the amplifier, our focus lies in reducing the contribution of transistor noise. In the conventional OTA without the source degeneration resistor, the transistor produces significant noise due to its substantial channel current. In contrast, our design utilizes the source-degenerated NMOS transistor, comprising a transistor and a source degeneration resistor, as illustrated in Figure 2. The noise generated by a source degeneration NMOS transistor primarily arises from the resistor, resulting in a significantly lower noise contribution compared to an MOS transistor operating at the same current level. Another benefit of employing source-degenerated NMOS transistors is that the noise induced by resistors is predominantly thermal noise, while NMOS transistors tend to produce a notable amount of 1/f noise unless they are sized with a considerably large area. In our neural amplifier, the input differential pair is composed of a pair of stacked large-area PMOS transistors, which is the major noise contributor of the amplifier. The PMOS transistors are chosen due to the fact that the 1/f noise of a PMOS transistor is one to two orders of magnitude lower than the 1/f noise of an NMOS transistor of the same size, as long as it does not significantly exceed the threshold voltage [17,18].

### 3.2. Maximizing G_m_ Analysis and Noise Analysis

**Figure 3:**
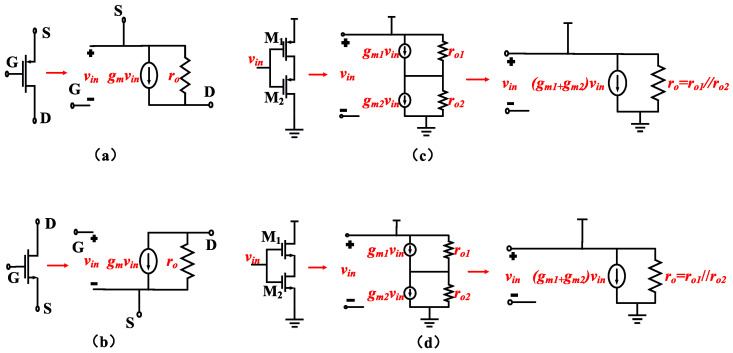
(**a**) Small-signal model of a PMOS transistor. (**b**) Small-signal model of an NMOS transistor. (**c**) Small-signal model of a PMOS transistor based on current reuse. (**d**) Small-signal model of an NMOS transistor based on current reuse.

To achieve low input-referred noise, it is crucial to maximize the transconductance (G_m_) of the OTA under a given total current. The maximum achievable G_m_ for an OTA is typically the transconductance of the PMOS transistor in the input differential pair, which we can refer to as g_m1_. Therefore, G_m_ ≈ g_m1_. Consequently, it is advantageous to operate the input transistors in the subthreshold region to maximize the g_m_ at a given current level. This implies that the input transistors need to have a larger W/L ratio. Based on this consideration, combined with Figure 3c,d, enhancing the input differential pair through the use of current reuse technology can increase the transconductance of the input differential pair without consuming additional current.

The total input-referred thermal noise can be approximately calculated by (2).

**Figure 4:**
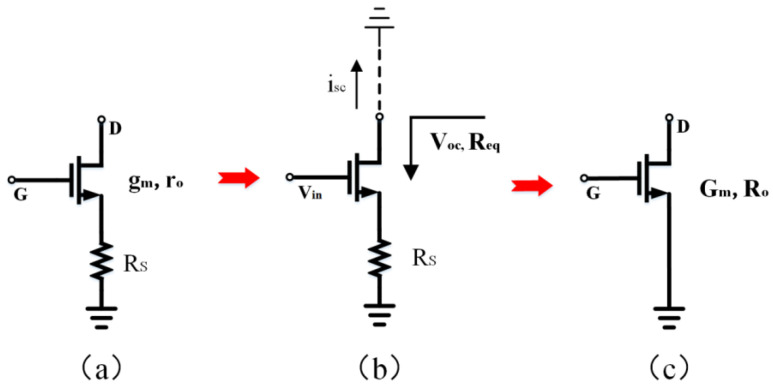
(**a**) An NMOS transistor with source degeneration. (**b**) An equivalent circuit is used to analyze NMOS transistor with source degeneration. (**c**) An NMOS transistor with source degeneration is equivalent to a single transistor with a smaller transconductance (G_m_) and larger output impedance (R_o_).

(2) Vin,thermal 2¯=[16kT3gm1(1+gm5gm1+gm7gm1+gm13gm1+gm15gm1)]Δf

where k is the Boltzmann constant, T is the absolute temperature, and g_m_ is the transconductance of its transistor. To reduce the total input-referred thermal noise, g_m5_, g_m7_, g_m13_, and g_m15_ must be significantly less than g_m1_ to minimize the noise contribution of the devices M_5_–M_8_ and M_13_–M_16_. After designing M_5_–M_8_ and M_13_–M_16_, g_m5_–g_m8_ and g_m13_–g_m16_ become the minimum. We can analyze M_5_–M_8_, M_15_, and M_16_ in combination with Figure 4.

Figure 4 illustrates the schematic diagram of the circuit used to determine the equivalent transconductance of a source-degenerated NMOS transistor. In Figure 4b, the open-circuit voltage (V_oc_), short circuit current (i_sc_), and equivalent resistance (R_eq_) are defined. Assuming a small signal current of zero enters the drain of the transistor, the resulting voltage on R_s_ is reduced to zero. This condition renders R_s_ independent of V_gs_ and V_oc_. Furthermore, the transistor’s equivalent resistance is increased by a factor of (1 + g_me_R_s_), where g_me_ represents the effective transconductance of the transistor (accounting for the body effect). Because i_sc_ = V_oc_/R_eq_, and V_oc_ is not influenced by R_s_, the i_sc_ decreases by the same factor as the output resistance increases. Considering the aforementioned properties, we can construct an equivalent transistor for an NMOS transistor with source degeneration, as depicted in Figure 4c. Including R_s_ in the circuit has an overall effect of increasing the output impedance (R_o_) and decreasing the equivalent transconductance (G_m_). By defining G_m_ and R_o_ and utilizing Equations (3), (5), and (6), we can ensure that the open-circuit voltage of the equivalent transistor remains unaffected by R_s_. Using this method, we can determine the equivalent transconductance of a source-degenerated NMOS transistor, as demonstrated in Equation (7), where R_o_ is equal to R_eq_.

(3) $$v_{\mathrm{oc}}={-g}_{m}r_{o}v_{\mathrm{in}}$$

(4) $$R_{\mathrm{eq}}=R_{S}+r_{o}+g_{\mathrm{me}}r_{o}R_{S}$$

(5) $$i_{\mathrm{sc}}=\frac{v_{\mathrm{oc}}}{R_{\mathrm{eq}}}=-\frac{g_{m}r_{o}}{R_{\mathrm{eq}}}v_{\mathrm{in}}$$

(6) $$G_{m}=-\frac{i_{\mathrm{sc}}}{v_{\mathrm{in}}}=\frac{g_{m}r_{o}}{R_{o}}$$

(7) $$G_{m}=\frac{g_{m}r_{o}}{R_{S}+r_{o}+g_{\mathrm{me}}r_{o}R_{S}}$$

According to Formula (7), the source degeneration transistor can result in a higher equivalent resistance (R_o_) and a lower transconductance (G_m_). This has significance in optimizing the input-referred noise of the amplifier.

Table 1 illustrates the operating points for transistors in the OTA. As shown in Table 1, by operating M_1_–M_4_ in the subthreshold region, we achieved a high g_m_/I_D_ ratio such that g_m1_ is much greater than g_m5_–g_m8_ and g_m13_–g_m16_, combining Figure 3, using current reuse technology to enhance the transconductance of the input transistors, with g_m1_ = g_mos1_ + g_mos3._ (The g_mos1_ is the transconductance of M_1_ and the g_mos3_ is the transconductance of M_3_).

As mentioned in Section 3.1, the 1/f noise (flicker noise) is also a key noise contributor in low-noise, low-frequency circuits. We mitigate the impact of flicker noise by using PMOS transistors as input devices and employing devices with large gate-source areas. The flicker noise is inversely proportional to the gate-source area, so all transistors should be made as large as possible to minimize the 1/f noise. However, as devices M_5_–M_8_ and M_13_–M_16_ are made larger, the total capacitance seen by the gate of M_5_–M_8_ and M_13_–M_16_ increase, and according to (1), when those transistors are made larger, C_p_ increases, and the total input-referred noise of the OTA also increases. To ensure noise minimization, there is an optimal size for M_5_–M_8_ and M_13_–M_16_. In our design, we decreased the size of M_5_–M_8_ and M_13_–M_16_ as much as possible, trading off the input-referred noise.

### 3.3. Noise Efficiency Factor

As mentioned in Section 1, the NEF proposed in [4] is adopted:

(8) $$NEF=V_{\mathrm{ni},\mathrm{rms}}\sqrt{\frac{2I_{\mathrm{tot}}}{\pi \cdot U_{T}\cdot 4kT\cdot BW}}$$

where V_ni,rms_ is the total input-referred rms noise voltage, I_tot_ is the total supply current, and BW is the −3 dB bandwidth of the amplifier in hertz, respectively.

The NEF limitation for MOSFET-based amplifiers stems from their current noise and maximum gm/I_D_ [19]. The input-referred rms noise of the ideal MOS transistor is expressed as

(9) $$V_{\mathrm{mos},\mathrm{rms}}=\sqrt{\frac{4kT\cdot \gamma \cdot g_{m}}{g_{m}^{2}}\cdot \frac{\pi }{2}\cdot BW}$$

where γ is the noise coefficient and g_m_ is the transconductance of an MOS transistor. When the transistor operates in the subthreshold region, we obtain g_m_ = κI_D_/U_T_, and the input-referred rms noise of the ideal MOS transistor [19] is expressed as

(10) $$V_{\mathrm{mos},\mathrm{rms}}=\sqrt{\frac{2kT\cdot U_{T}}{\kappa^{2}I_{D}}\cdot \frac{\pi }{2}\cdot BW}$$

The theoretical limit of the NEF of an OTA that uses a differential pair as an input stage is when the two differential pair transistors are the only noise sources in the circuit. The input-referred noise of the OTA is then $\bar{V_{ni,rms}^{2}}=2\times \bar{V_{mos,rms}^{2}}$.

Assuming a first-order roll-off of the frequency response, the input-referred rms noise of the ideal OTA is expressed as

(11) $$V_{\mathrm{ni},\mathrm{rms}}=\sqrt{\frac{4kT\cdot U_{T}}{\kappa^{2}I_{D}}\cdot \frac{\pi }{2}\cdot BW}$$

Combining (8) and (11), we obtain the theoretical limit for the NEF of any OTA that uses a subthreshold MOS differential pair to be

(12) $$NEF=\sqrt{\frac{I_{\mathrm{tot}}}{\kappa^{2}\cdot I_{D}}}$$

Assuming a typical value of κ = 0.7 and as mentioned in Section 3.1, a 1:10 current scaling ratio is employed to lower the power consumption of the amplifier. Consequently, the total current consumption of the first stage amplifier is equivalent to 2.2 times I_B._ Therefore, I_tot_ = 2.2 I_D_. We can conclude that the theoretical limit value of the NEF is 2.12.

## 4. Detailed Circuit Implementation

**Figure 5:**
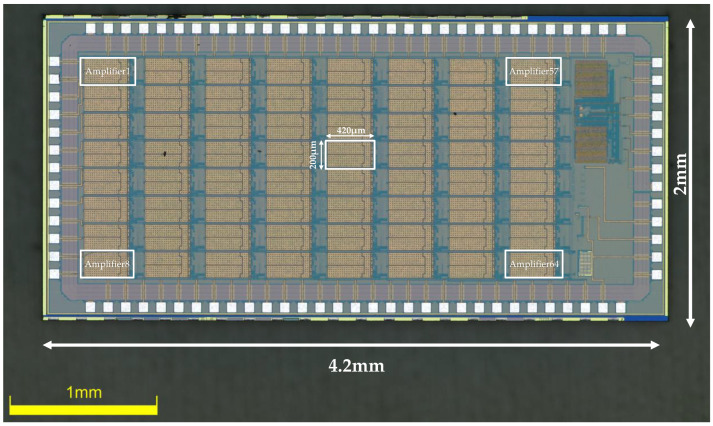
Die microphotograph of the proposed neural recording amplifier ASIC.

The amplifier was fabricated in the TSMC 0.18μm CMOS 1P6M process. All the source degeneration resistors are constructed using high-resistance polysilicon, with a resistance value of 186 KΩ. Metal–Insulator–Metal (MIM) capacitors are used for C_in_ and C_f_, which offer high-precision capacitance for accurately defining the closed-loop gain of the amplifier. By setting the value of C_in_ to 20 pF and C_f_ to 200 fF, the first stage is designed to provide a gain of approximately 100 (40 dB). The second stage offers a controllable gain of x2 and x1, thus setting the total gain of the amplifier to be ×200 and ×100, the total gain adjustable (×200, ×100). Each amplifier occupies active silicon the area of 0.082 mm^2^. An on-chip bandgap reference circuit generates all the reference currents and voltages for the entire chip to minimize the use of off-chip components. A chip microphotograph of the amplifier is shown in Figure 5 (the chip measures 2 mm × 4.2 mm, and contains 64 channels of a low-noise, low-power neural amplifier, a 64 to 1 MUX, a bandgap reference, and an ADC buffer).

## 5. Measurement Results

Each channel of the amplifier consumes 3.8 μA from a 1.8 V supply, which can be broken down as follows. The first-stage OTA consumes 3.6 μA, and the second-stage VGA consumes 0.2 μA. We do not include the bias current (1 μA), since it can be shared by many amplifiers in the array.

**Figure 6:**
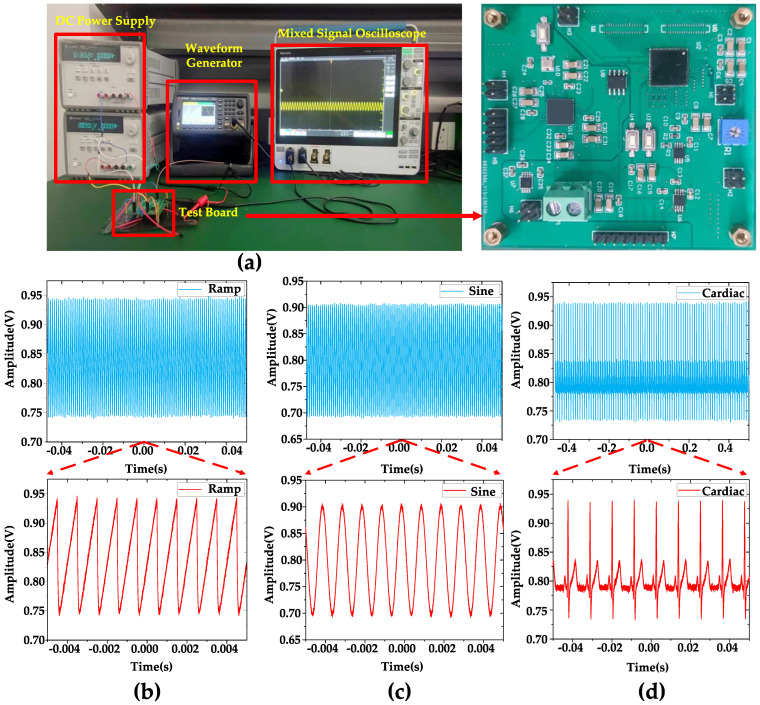
(**a**) Test equipment and test board. (**b**) DC measurement when inputting a 1 mVpp, 1 kHz ramping signal. (**c**) DC measurement when inputting a 1 mVpp 1 kHz sine signal. (**d**) DC measurement when inputting a 1 mVpp 1 kHz artificial cardiac signal. The blue part is a long period of waveform, and the red part is a part of waveform captured from it for display. When sin signal/ramp signal/artificial cardiac signal is input, the output signal of the amplifier is the sin signal/ramp signal/artificial cardiac signal amplified according to the scale.

Figure 6 displays the equipment used for the measurements, including the test board, along with the observed waveforms. Figure 6b–d show that when inputting 1 mVpp, 1 kHz ramp, sine, and artificial cardiac signals generated by the Keysight 33600 A true waveform generator, the DC measurement of the output waveform is performed using a Tektronix MSO54 Mixed Signal Oscilloscope. As mentioned in Section 1, the DC offset is an issue to be considered in a neural signal amplifier. Since the reference voltage of the amplifier is 0.9 V, it is expected that the output waveform of the amplifier will exhibit fluctuations above and below 0.9 V. Therefore, conducting DC measurements can serve as a means to verify this behavior.

**Figure 7:**
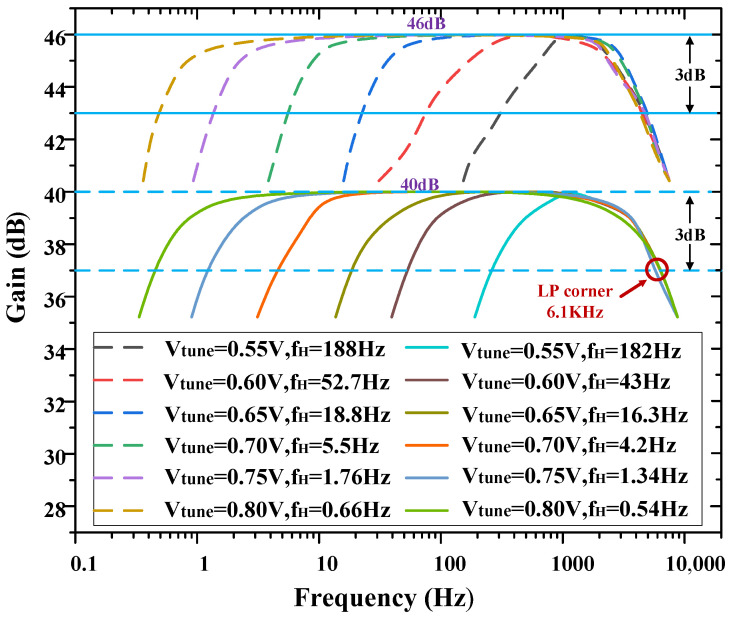
Measured frequency response of the neural recording amplifier with tunable high−pass corner frequency.

As mentioned in Section 1, taking into account the characteristics of the LFPs and APs, the −3 dB bandwidth of the amplifier should be designed to capture a wide range of neural signals. To achieve this, the high-pass corner frequency of the amplifier can be adjusted to 0.54 Hz, allowing for the recording of low-frequency signals. Additionally, a load capacitor of 8 pF was chosen to establish the low-pass corner frequency of the amplifier at 6.1 kHz, enabling the inclusion of high-frequency signals within the bandwidth. Figure 7 shows the AC frequency response of one channel of the overall amplifier. The amplifier has a measured low-pass cut-off frequency of 6.1 kHz, and its high-pass cut-off frequency is tunable from 0.54 Hz to 182 Hz by V_tune_, the voltage of V_tune_ is regulated by a potentiometer.

**Figure 8:**
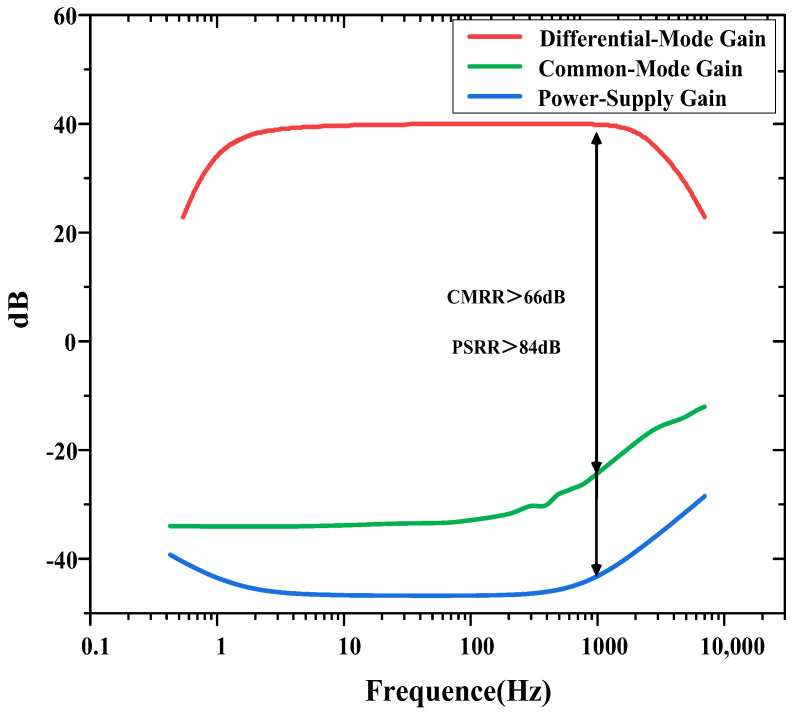
CMRR and PSRR measurements of the neural recording amplifier.

The measured CMRR and PSRR are shown in Figure 8. The CMRR is calculated as the ratio of the differential-mode gain to the common-mode gain. The PSRR is calculated as the ratio of the differential-mode gain to the gain from the power supply to the output. The measured CMRR and PSRR exceed 66 and 84 dB at 1 kHz, respectively.

**Figure 9:**
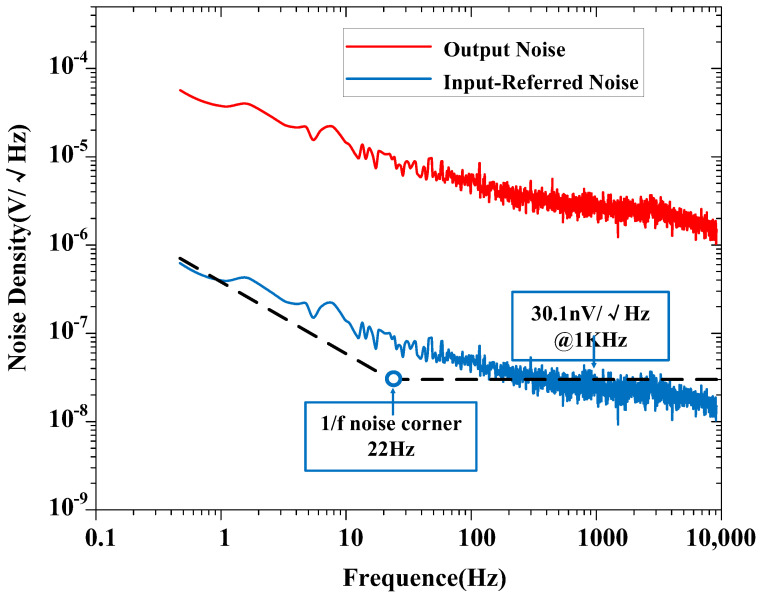
Measured output noise and input-referred noise spectrum of the proposed amplifier (at a gain of 100).

**Figure 10:**
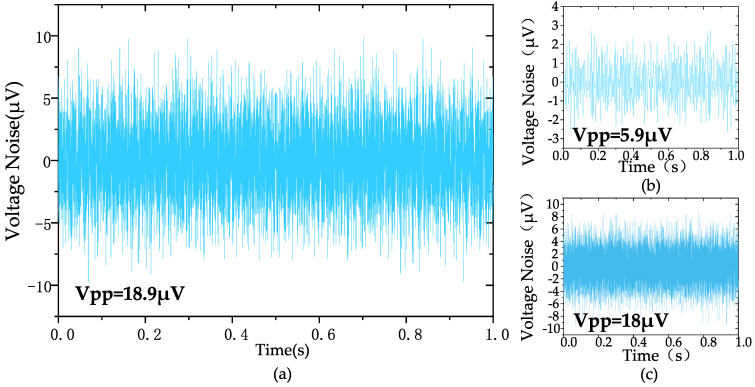
The measured transient input-referred noise waveform: (**a**) 1 Hz–6.1 kHz, (**b**) 1 Hz–200 Hz, (**c**) 200 Hz–6.1 kHz.

The measured input-referred noise spectrum of the amplifier is shown in Figure 9, which is obtained by dividing the output noise spectrum by the mid-band gain of the amplifier (at a gain of 100). The 1/f noise corner of the design was found to be roughly 22 Hz. The measured transient input-referred noise waveform is shown in Figure 10. Figure 10a records the input-referred peak-to-peak noise voltage in the frequency range 1 Hz to 6.1 kHz; the total input-referred rms noise is 3.1 μVrms integrated from 1 Hz to 6.1 kHz. The measured integrated noise is 0.96 and 2.95 μVrms in the frequency band of 1–200 Hz and 0.2 k–6.1 kHz, respectively. An input-referred peak-to-peak voltage noise of 5.9 μVpp (1–200 Hz) and 18 μVpp (0.2 k–6.1 kHz) are measured, as shown in Figure 10b,c, respectively. By using (9), the NEF of the amplifier is calculated to be 2.97 from the measurement results.

The power efficiency factor (PEF) that includes the supply voltage VDD is also an important parameter for evaluating the power efficiency for biomedical amplifiers. The PEF can be calculated as

(13) $$PEF=NEF^{2}\cdot \frac{P_{\mathrm{tot}}}{I_{\mathrm{tot}}}=NEF^{2}\cdot VDD$$

And the PEF of the amplifier is calculated to be 10.17.

**Figure 11:**
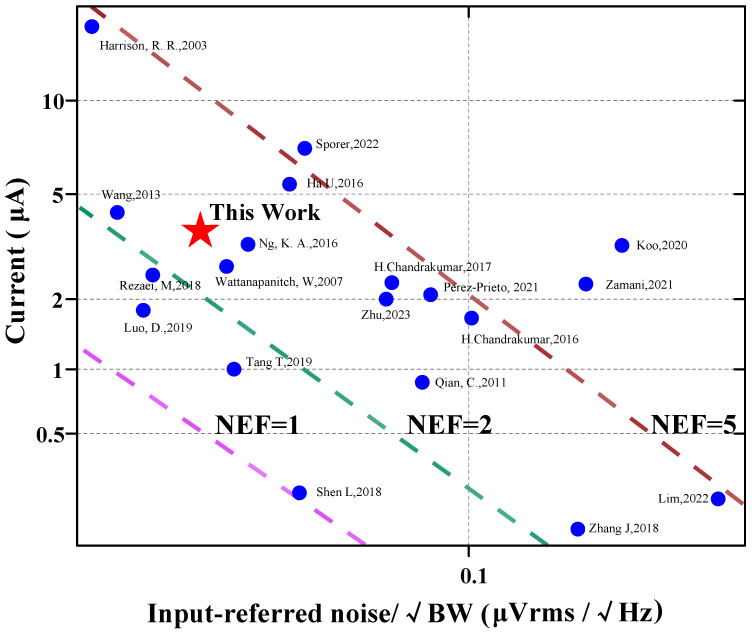
Comparison with the existing amplifier designs of the input-referred noise versus the supply current of the amplifier (references: [6,7,9,13,15,16,17,20,21,22,23,24,25,26,27,28,29,30,31]). These colored slashes represent the value of NEF, such as the pink line, where the value of NEF is 1, the area below the slash is NEF < 1, and the area above the slash is NEF > 1. The green and brown lines work the same way. For example, for the work of Tang, T, 2019, the NEF value of this work is below NEF = 2 (green line) and above NEF = 1 (pink line), which can show that its NEF value is between 1–2. The position of each work point in the picture is based on the current consumed by its design. The resulting −3 dB bandwidth and the input referred noise.

Figure 11 [6,7,9,13,15,16,17,20,21,22,23,24,25,26,27,28,29,30,31] shows the input-referred noise versus the supply current of the amplifier. The proposed work features a low input-referred noise while achieving a competitive NEF. Table 2 compares the proposed work with state-of-the-art designs in the literature. Three different topologies of AFEs are compared. Although [20] and [32] achieved impressive NEF (Noise Efficiency Factor) values of 1.07 and 0.86, respectively. In [20], a NEF value of 1.07 was obtained by stacking three gm cells. On the other hand, [32] utilized five differential pairs with AC-coupled inputs to achieve an NEF value of 0.86. Such aggressive stacking of g_m_ cells results in limited headroom for each transistor. Typical amplifier designs are currently used in the industry, such as the CCIA [17] and Chopper [33] structures, as well as existing applications in the field of BMI aiming for high-resolution and high-density neural probes like Neuralpixels [34,35]. The design offers several advantages. Firstly, it occupies a smaller area compared to other designs, allowing for the efficient use of limited chip real estate. Additionally, the design achieves a smaller input-referred noise, leading to improved signal quality. Moreover, it provides a larger range of −3 dB bandwidth, enabling the recording of a wider range of signals. Furthermore, the design exhibits relatively low power consumption, making it energy-efficient. Lastly, the NEF and PEF of the design are also superior under the 0.18 μm CMOS process.

## 6. Conclusions

In this paper, a low-noise and low-power amplifier with a CCIA topology is proposed for neural signal acquisition. The amplifier reduces input-referred noise by stacking two PMOS transistors in combination with source degeneration resistor technology, rather than stacking multiple g_m_ cells that consume headroom for each transistor. And the current scaling technology is used to reduce the power consumption of the amplifier. Different from the traditional current scaling technology, this design uses two separate NMOS transistors to divide the current, so as to achieve current scaling. In contrast to the traditional approach, which requires additional bias current branches, this design method is more energy efficient. The design was fabricated using the TSMC 0.18 μm MS RF G process. The measurement results demonstrate the amplifier’s favorable power and noise performance. The measured −3 dB bandwidth of 0.54 Hz–6.1 kHz indicates its capability to record LFPs and APs. This architecture is well suited as a front-end amplifier for power-constrained or energy-sensitive applications, particularly in the field of biomedical implants.

## Figures and Tables

**Table 1:** Operating points for transistors in the OTA.

Devices	I_D_ (μA)	g_m_ (μs)	g_m_/I_D_	Operating Region
M_1_, M_2_	1.6	41.95	26.2	Sub-threshold
M_3_, M_4_	1.6	43.12	27	Sub-threshold
M_5_, M_7_, M_6_, M_8_	0.16	2.16	13.5	Strong inversion
M_9_, M_10_	0.16	3.89	24.3	Sub-threshold
M_11_, M_12_	0.16	4.12	25.7	Sub-threshold
M_13_, M_14_	0.16	1.2	7.5	Strong inversion
M_15_, M_16_	1.44	8.53	5.9	Strong inversion

**Table 2:** Performance and comparison of the proposed neural amplifier.

	[13]	[17]	[19]	[22]	[28]	[31]	[33]	[34]	[35]	[36]	This Work
Technology (μm)	0.6	1.5	0.35	0.18	0.18	0.18	0.8	0.18	0.13	0.04	0.18
CMRR (dB)	>66	>83	>65	110	>103	75	>100	60	60	107	66
PSRR (dB)	>80	>85	>70	-	84	68	-	76	70	>70	84
Input-referred noise (μVrms)	3.07 (0.5 Hz–30 kHz)	2.2	2.05 (0.1 Hz–10 kHz)	4.2 (1 Hz–10 kHz)	7.5	2.1 (1 Hz–200 Hz)	0.98 (0.05 Hz–100 Hz)	3.2	6.36	1.7 (1 Hz–260 Hz)	3.1 (1 Hz–6.1 kHz)
Bandwidth (Hz)	0.36–1300	0.025–7200	0.2–200	HP: 0.15/0.26 LP: 9400/12,100	10–10,000	0.9–900	0.05–180	0.5–6000	0.3–10,000	HP: 0.2–550 LP: 260–3800	0.54–6100
Gain (dB)	39.4	39.5	39.8	60/54	46	80	50.5/41	29.5–72	68	37	46/40
Current (μA) & Power (μW)	0.872 & 2.4	16 & 80	0.16 & 0.32	1 & 1	2 &3.6	2.3 & 2.3	1 & 2	3.9 & 7.02	40.9 & 49	2.06 & 2.47	3.8 & 6.84
NEF	3.09	4	2.26	1.7	6.27	8.6	4.6	3.08	3.8	4.1	2.97
PEF	26.7	80	10.2	2.89	20.3	8.6	38.1	17.13	17.33	20.17	10.17
Area (mm²)	0.13	0.16	0.18	0.09	0.07	0.051	0.8	0.19	0.12	0.048	0.082
Topology	CCIA	CCIA	CCIA	CCIA	DDA	Chopper	Chopper	CCIA	CCIA	Chopper	CCIA

## Data Availability

Data are contained within the article.
